# Characterization of T-cell immune responses against *Staphylococcus chromogenes* naturally-induced subclinical mastitis in dairy cows

**DOI:** 10.3389/fimmu.2025.1679963

**Published:** 2025-10-10

**Authors:** Chen Yu, Jie Wu, Sheng Zhang, Chunmei Ye, Guojun Wei, Zhangping Yang, Zhipeng Zhang

**Affiliations:** ^1^ College of Animal Science and Technology, Yangzhou University, Yangzhou, China; ^2^ Key Laboratory of Dairy Cattles Genetic Improvement in Southern China Ministry of Agriculture and Rural Affairs, Bright Farming Co., Ltd., Shanghai, China; ^3^ Hebei Pinyuan Biotechnology Co., Ltd, Shijiazhuang, China

**Keywords:** subclinical mastitis, T-cell response, Staphylococcus chromogenes, flow cytometry, cow

## Abstract

**Introduction:**

Subclinical mastitis in dairy cows is an asymptomatic inflammatory condition of the mammary gland during lactation. It is frequently associated with reduced milk yield, impaired milk quality, and an elevated risk of progression to clinical mastitis. The dysregulation of immune homeostasis characterizing this subclinical state represents a significant yet often overlooked concern in dairy herds.

**Methods:**

We characterized T cell subsets in cows with *Staphylococcus chromogenes*-induced subclinical mastitis and healthy controls.

**Results:**

We found that *S. chromogenes* infection induced an increase in the percentage of γδ T cells and a decrease in CD4^+^CD8^+^ double-positive (DP) T cells within milk cells, while significantly elevating the percentage of γδ T cells expressing CD44. Cell sorting combined with quantitative RT-PCR has indicated that infection by *S. chromogenes* may activate γδ T cells. Specifically, in subclinical mastitis milk, the expression levels of five cytokines—IFN-γ, IL-2, TNF-α, IL-17, and GZMB—were significantly higher in WC1^+^ γδ T cells compared to healthy cows. Furthermore, these cells released elevated levels of PRF1, which directly killed target cells infected with bacteria compared to the levels observed in healthy cows.

**Discussion:**

Our results reveal, for the first time, the significant role of γδ T cells in *S. chromogenes* -induced subclinical mastitis, providing new insights into the immune protective mechanisms against subclinical mastitis in cows.

## 1 Introduction

Mastitis is one of the most costly and most prevalent diseases in dairy farming, with significant zoonotic potential. The disease manifests as either clinical or subclinical mastitis. Among these, subclinical mastitis is the predominant and more widespread form in global dairy herds, causing economic losses three to four times higher than clinical mastitis (1). Cows with subclinical mastitis face an increased risk of developing clinical mastitis, which may lead to more severe symptoms, compromised welfare, higher veterinary costs, and even premature culling (2, 3). Multiple bacterial species may cause subclinical mastitis. However, *Staphylococcus chromogenes (S. chromogenes)* constitute the predominant etiological agents, accounting for significant economic losses in dairy operations (4).

*S. chromogenes* is a coagulase-negative, Gram-positive coccus primarily colonizing the skin, mammary glands, and mucous membranes of livestock (e.g., cattle, pigs, horses) (5–7). This bacterium is frequently isolated from bovine milk and carries multiple antimicrobial resistance genes (8, 9),. It can also inhibit the growth of other major clinical mastitis-causing bacteria *in vitro (* 10). However, epidemiological data indicate that it does not exhibit uniform pathogenicity across all hosts. For instance, *S. chromogenes* has been implicated in clinical mastitis in dairy cows (11), exudative epidermitis in pigs (12), and even bloodstream infections in immunocompromised humans (13)—suggesting its virulence may be dependent on host immune status. Collectively, these findings highlight that although *S. chromogenes* is predominantly reported as a common pathogen in bovine subclinical mastitis with limited human cases, it may still transmit to humans via the food chain (e.g., milk) or environmental exposure, potentiating the risk of antimicrobial-resistant infections.

Subclinical mastitis induced by *S. chromogenes* features persistent infection but lacks overt clinical signs in the udder or milk. It is detected primarily through an elevated somatic cell count (SCC) in the milk, signifying a host immune response to the infection. Nevertheless, this specific immune response has not yet been sufficiently studied (14). T cells are pivotal in the bovine mammary immune response, contributing through direct cytotoxic elimination of pathogens, activation of macrophages via cytokine secretion, and recruitment of neutrophils. A recent *in vitro* study investigating the proliferative response of blood-derived T lymphocytes to bovine-associated staphylococci found that *Staphylococcus aureus* strains causing persistent intramammary infections triggered proliferation in both CD4^+^ and CD8^+^ T cells. Furthermore, these strains significantly increased IL-17A and IFN-γ production by peripheral blood mononuclear cells (PBMCs). However, the activation profile and memory characteristics of T cells involved in this persistent infection remain incompletely understood (15).

Thus, we defined a cohort of dairy cows with chronic subclinical mastitis caused by natural Staphylococcus chromogenes infection in this study. Our analysis focused on the distribution, activation profiles, and memory signatures of their T cell subsets. Understanding these features may reveal the reasons underlying the elevated clinical mastitis risk observed in these cows and contribute to designing more effective on-farm veterinary management plans.

## 2 Materials and methods

### 2.1 Dairy cows

All the experimental methods in this study were evaluated and approved by the Experimental Animal Ethics Committee of Yangzhou University (NO. 202503247). The environmental conditions of this facility meet the requirements for standard animal experimentation facilities outlined in the Chinese National Standard Experimental Animal Environment and Facilities (GB14925-2023). Animal husbandry management and experimental procedures are conducted in compliance with all applicable Chinese regulations.

The cows in this study originated from a well-managed commercial dairy farm. Sixty cows with comparable age, body condition, milk yield, and SCC were carefully selected. Forty cows had been professionally diagnosed with subclinical mastitis by veterinarians using the Lanzhou Mastitis Test (LMT) (16) and had been experiencing the condition for 15 to 30 days, while the remaining twenty cows were healthy. Peripheral blood samples (5 ml per cow) were aseptically collected from all selected cows at a single time point into EDTA-anticoagulated vacuum tubes. For milk sample collection, teats were washed, dried using paper towels, and disinfected with 70% alcohol swabs. The initial four milk streams were discarded to minimize contamination, followed by collection of 500 ml milk into a sterile container. Samples were immediately stored in a cooler box with ice packs and processed within 3 hours.

### 2.2 Microbiological analysis

To facilitate bacterial identification, milk samples underwent serial 10-fold dilutions until plates contained <20 colonies. When multiple colony morphologies appeared on a plate, an additional sub-culture was performed to isolate pure strains. Following DNA extraction, bacterial 16S rDNA genes were amplified using universal primers 27F (5’-AGAGTTTGATCCTGGCTCAG-3’) and 1492R (5’-GGTTACCTTGTTACGACTT-3’) (17). PCR products were submitted to Qingke (Shanghai, China) for 16S rRNA gene sequencing. Bacterial species were identified via BLASTN analysis of assembled sequences against the NCBI database. For isolates identified as *S. chromogenes*, phylogenetic analysis was conducted using MEGA 7 software (Kumar et al., 2018). Pure cultures were maintained in brain-heart infusion broth (Cloolaber, Beijing, China) at 37 °C and preserved long-term at -80 °C in 30% glycerol.

### 2.3 Peripheral blood mononuclear cell separations

Peripheral blood mononuclear cells (PBMCs) were isolated from anticoagulated blood of cows infected solely with *S. chromogenes* and healthy control cows using density gradient centrifugation (650 × g, 35 min) with lymphocyte separation medium. Purified PBMCs were resuspended in complete medium (RPMI-1640 supplemented with 10% fetal bovine serum and 1% antibiotics) and immediately processed for multiparametric flow cytometry analysis.

### 2.4 Phenotypic analysis of T cells and cell sorting

Freshly isolated peripheral blood mononuclear cells (PBMCs) were resuspended in FACS buffer (PBS containing 0.5% fetal bovine serum) and adjusted to a concentration of 2 × 10^6^ cells per sample for T-cell subset phenotyping. The monoclonal antibodies and secondary antibodies used for cell surface staining are listed in **Supplementary Table S2**. Cells were stained in 96-well V-bottom plates, followed by incubation with 1% bovine serum at 37 °C for 20 minutes to block Fc receptors (Hao et al., 2021). After centrifugation at 1,600 rpm for 5 min at 4 °C, cells were washed twice with FACS buffer. A minimum of 1 × 10^5^ cells were acquired for flow cytometric analysis using a CytoFLEX S flow cytometer (Beckman Coulter, China) equipped with four lasers (488 nm, 638 nm, 405 nm, and 561 nm) and an HTS (High Throughput Sampler) loader. Automatic compensation was applied based on single-stained controls. Fluorescence-minus-one (FMO) controls were used to establish gating strategies (**Supplementary Figure S1**). Data analysis was performed using FlowJo software (v10.8.1), and results were exported to Prism 9.4 (GraphPad Software) for statistical analysis and graphical representation.

Automatic compensation was implemented in the sorting experiments by employing CD3 and WC1 to denote distinct fluorochromes, ensuring demarcated positive and negative populations for each fluorochrome color in comparison to unstained control samples. This facilitated the computation of compensation through software. The sorting process utilized an 85μm nozzle, with a rate not exceeding 2,000 events per second. Post-sorting analysis was conducted using FlowJo software. (**Supplementary Figure S2**).

### 2.5 Quantitative PCR

Total RNA was extracted from cells using an RNA-easy Isolation Reagent (Vazyme, Nanjing, China) according to the manufacturer’s instruction. The obtained RNA quality was assessed using a 2100 bioanalyzer (Agilent Technologies, Santa Clara, CA, USA), and the RNA quality number (RQN) was >7.0 for all samples. cDNA was synthesized by HiScript III RT SuperMix (R323-01, Vazyme, China), and AceQuniversal SYBR Master Mix for qPCR was used in the qRT-PCR experiment (Vazyme, China) on an ABI QuantStudio PCR system (Applied Biosystems, Foster City, CA, USA). Internal control genes including GAPDH were selected for normalization of each target gene’s expression by using geometric mean. The 2^−ΔΔCt^ approach was utilized for relative quantification.

### 2.6 Statistical analyses

Statistical significance was calculated using independent samples t-tests in SPSS software (version 20.0). Data are presented as comparisons between the healthy group and the subclinical mastitis group. Results were considered statistically significant when *P* < 0.05 (*), *P* < 0.01 (**), or *P* < 0.001 (***).

## 3 Results

### 3.1 *S. chromogenes* found in milk samples from cows with subclinical mastitis

**Figure 1:**
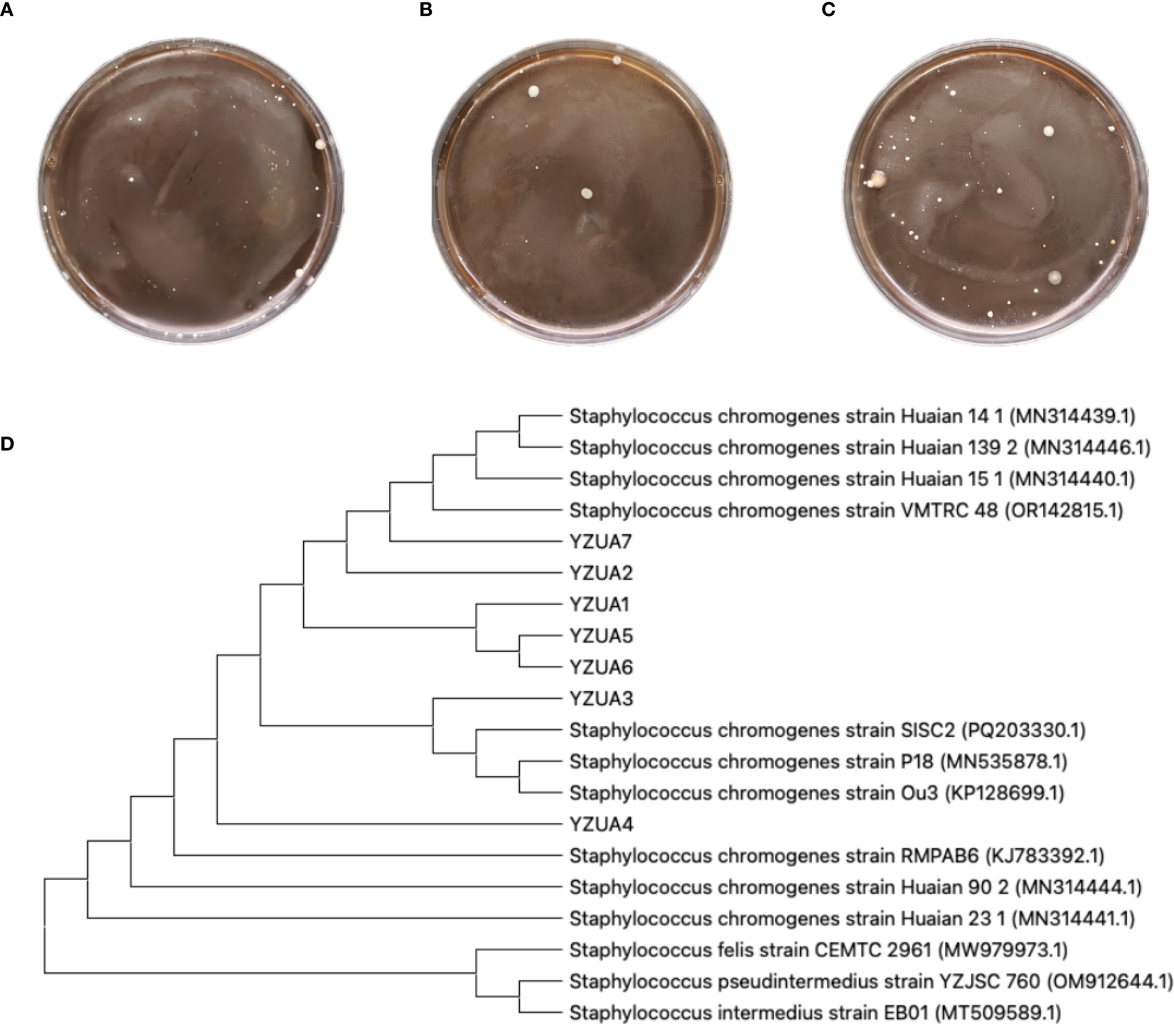
Isolation and identification of pathogenic bacteria. **(A)** Representative plates from undiluted milk samples from healthy cows. **(B)** Representative plates from milk samples with subclinical mastitis. **(C)**Representative plates from milk samples with mastitis. **(D)** Phylogenetic tree analysis of *S. chromogenes*. The YZU font denotes *S. chromogenes* strains isolated in this study. Strains MN314441.1 and MN314444.1 were derived from healthy cattle isolates, strain MN535878.1 originated from subclinical mastitis cattle, strains MN314440.1, MN314446.1, and MN314439.1 were isolated from clinical mastitis cattle, and strain KP128699.1 was obtained from an insect.

There were small amount of bacterial colonies on culture dishes plated with milk from 20 healthy cows milk (**Figures 1A**). These bacteria were identified as non-mastitis-causing environmental bacteria in normal circumstances, including *Bacillus licheniformis*, *Alcaligenes faecalis* and *Alfalfa rhizobium*. Milk samples from 20 cows with subclinical mastitis yielded smooth, opaque, cream-colored colonies with entire margins (**Figure 1B**), presumptively identified as *S. chromogenes*. These 20 milk samples, seven presumptive isolates (designated YZU1-7) were obtained. BLAST analysis of their sequencing data revealed >98% similarity with *S. chromogenes* strains in the GenBank database. In the milk samples from the remaining 20 cows with subclinical mastitis, besides *S. chromogenes*, other established mastitis pathogens including *Staphylococcus aureus* and *Escherichia coli* were detected. Therefore, these 20 cows were excluded from subsequent studies (**Figure 1C**). Phylogenetic reconstruction analysis revealed that strains YZU1, YZU2, YZU3, YZU5, YZU6, and YZU7 clustered into a monophyletic clade, while YZU4 exhibited the closest genetic relationship with *S. chromogenes* strain KJ783392.1 (**Figure 1D**). All seven isolates demonstrated significant genetic differentiation from closely related species such as *Staphylococcus felis*, *Staphylococcus pseudintermedius*, and *Staphylococcus intermedius*, confirming their taxonomic classification as *S. chromogenes*. These seven YZU strains were positioned between isolates from subclinical and clinical mastitis cases.

### 3.2 *S. chromogenes*-induced subclinical mastitis elevated γδ T-cell frequency in milk but did not alter αβ T-cell frequency

**Figure 2:**
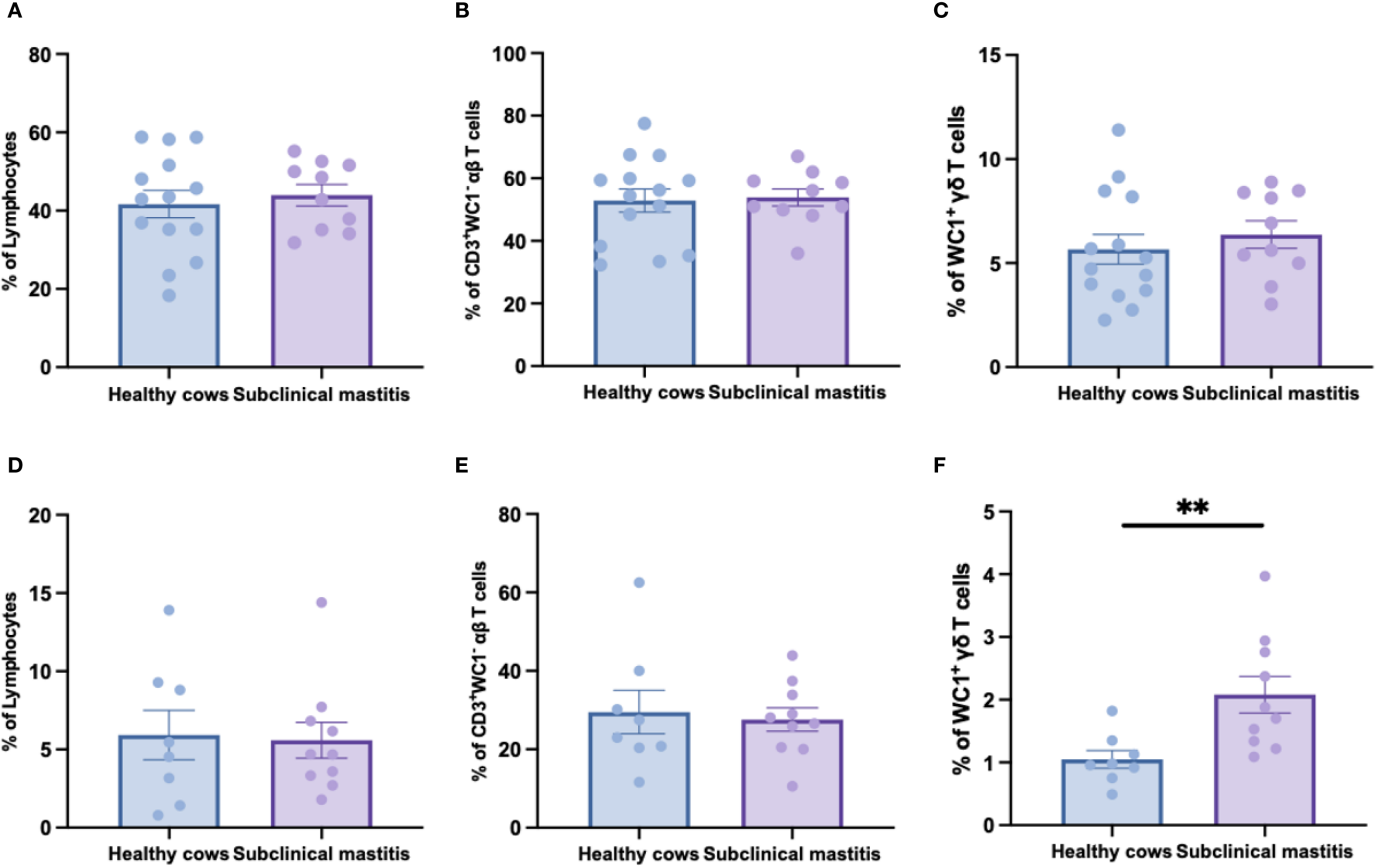
The distribution of αβ and γδ T cells in the peripheral blood and milk of healthy and subclinical mastitis cows. The percentage of lymphocytes **(A)** in PBMC and the percentages of αβ **(B)** and γδ **(C)** T cells within the lymphocytes. The percentage of lymphocytes **(D)** in the cells of milk as well as the percentages of αβ **(E)** and γδ **(F)** T cells among this group of lymphocytes. Results were considered statistically significant when P < 0.01 (**).

To elucidate the T-cell characteristics of *S. chromogenes*-induced subclinical mastitis, flow cytometric analysis was performed on peripheral blood and milk samples from healthy and affected cows. Surprisingly, no significant differences were observed in the frequencies of αβ T-cells and γδ T-cells in peripheral blood between healthy and subclinical mastitis cows (**Figures 2A–C**). Notably, while αβ T cell proportions in milk samples were similar across groups, the affected cows exhibited a pronounced expansion of γδ T cells when compared to healthy controls (**Figures 2D–F**). This infection-site-specific expansion of γδ T-cells highlights their pivotal role in innate immune responses during subclinical mastitis. The localized accumulation of these cells in milk suggests their potential as sensitive, non-invasive immunological markers for subclinical mastitis detection.

### 3.3 *S. chromogenes*-induced subclinical mastitis reduced only CD8^+^CD4^+^ DP T cells frequency in αβ T cells from cows milk

**Figure 3:**
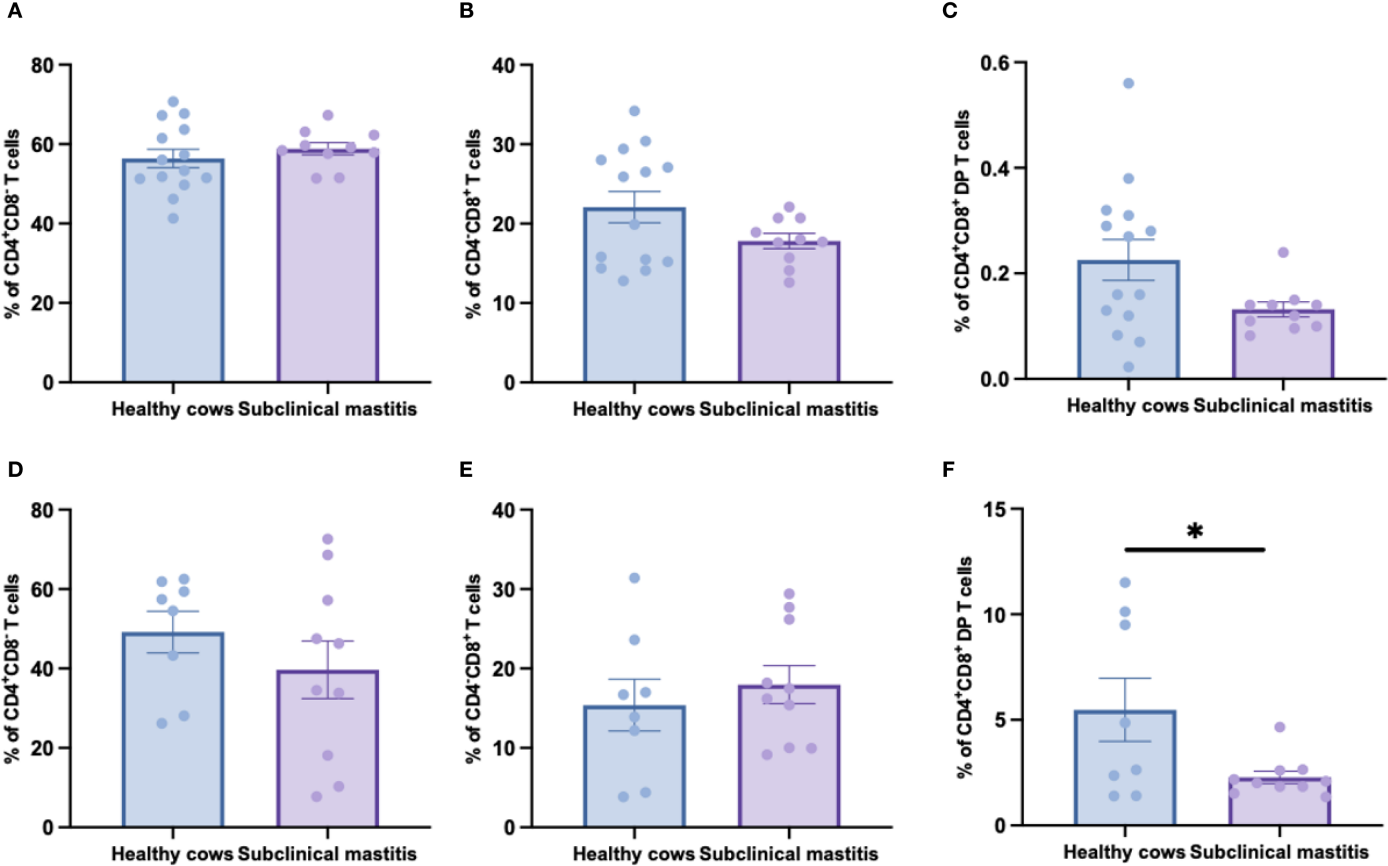
The distribution of CD4^+^, CD8^+^ and CD8^+^CD4^+^ DP T cells in αβ T cells of healthy and subclinical mastitis cows. The percentages of CD4^+^ **(A)**, CD8^+^ **(B)** and CD8^+^CD4^+^ DP **(C)** T cells in αβ T cells from PBMCs. The percentages of CD4^+^ **(D)**, CD8^+^ **(E)** and CD8^+^CD4^+^ DP **(F)** T cells in αβ T cells from milk. Results were considered statistically significant when P < 0.05 (*).

Since no significant differences in αβ T cells frequencies between healthy and subclinical mastitis samples in both peripheral blood and milk, we further delineated three subsets within αβ T cells using CD4 and CD8 antibodies to analyze the distribution patterns of CD4^+^, CD8^+^, and CD4^+^CD8^+^ double-positive (DP) T cells subsets. Peripheral blood analysis (**Figures 3A–C**) revealed no significant differences in the frequencies of CD4^+^, CD8^+^, and double-positive T cells among the groups, indicating that the homeostasis of αβ T cells subsets in systemic circulation was maintained during subclinical mastitis. Conversely, milk analysis uncovered distinct immunopathological features: while CD4^+^ and CD8^+^ T cell frequencies remained comparable, DP T-cells proportions were significantly reduced in affected cows (**Figures 3D–F**). This suggests that the exhaustion of DP T-cells in subclinical mastitis may represent a critical factor predisposing to progression into clinical mastitis.

### 3.4 Significant increase in CD44 expression of γδ T-cells in subclinical mastitis milk compared to healthy cows milk

**Figure 4:**
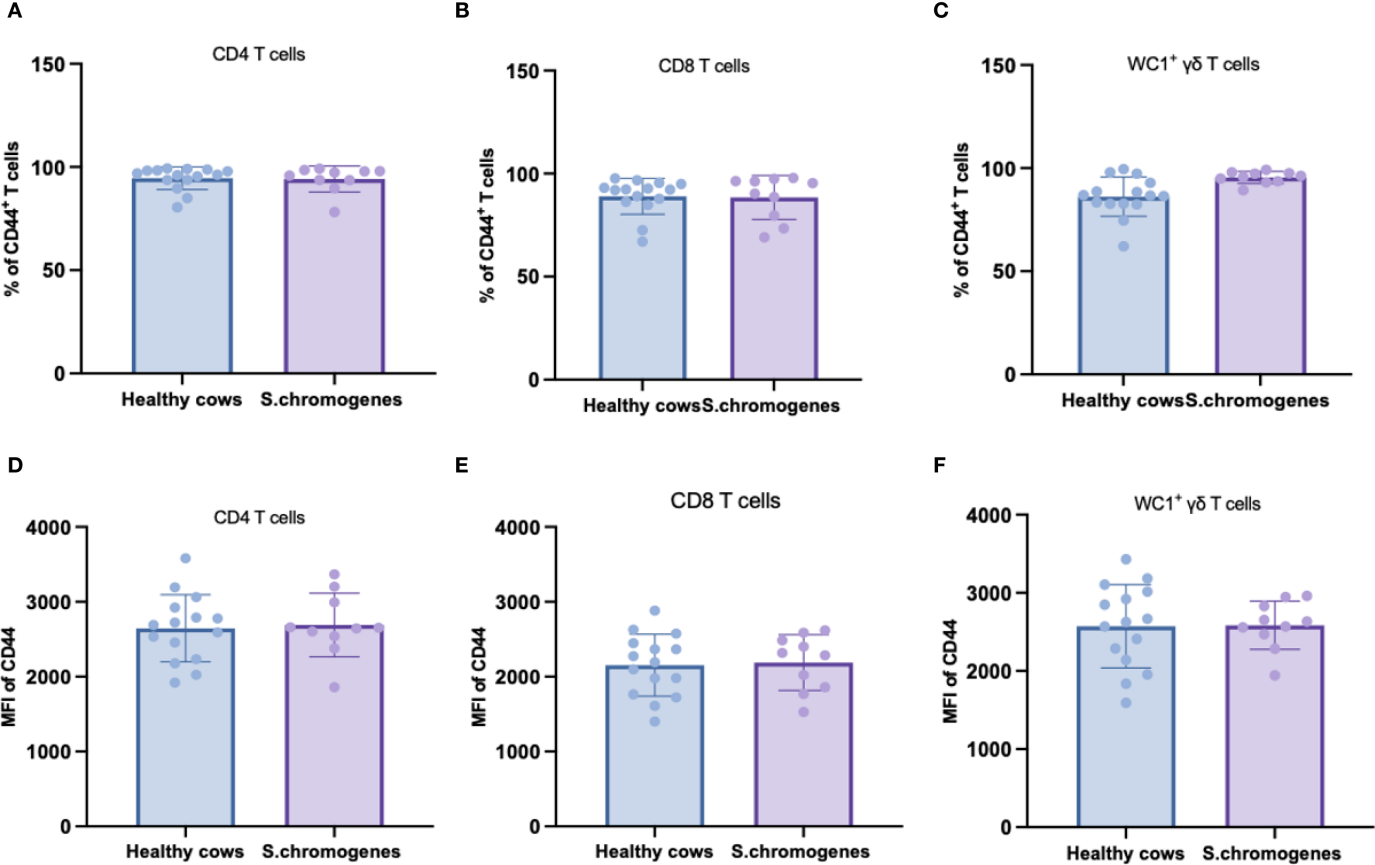
Difference of CD44 expression in different subsets of T cells (CD4^+^, CD8^+^, WC1^+^) in the peripheral blood of healthy and subclinical mastitis cows. Representative histograms show CD44 expression on CD4^+^CD8^-^ **(A)**, CD4^-^ CD8^+^ **(B)** T cells and WC1^+^ γδ T cells **(C)** from the PBMCs. Bar graphs show MFI of CD44 on CD4^+^ **(D)**, CD8^+^ T cells **(E)** and WC1^+^ γδ T cells **(F)** from the two groups.

**Figure 5:**
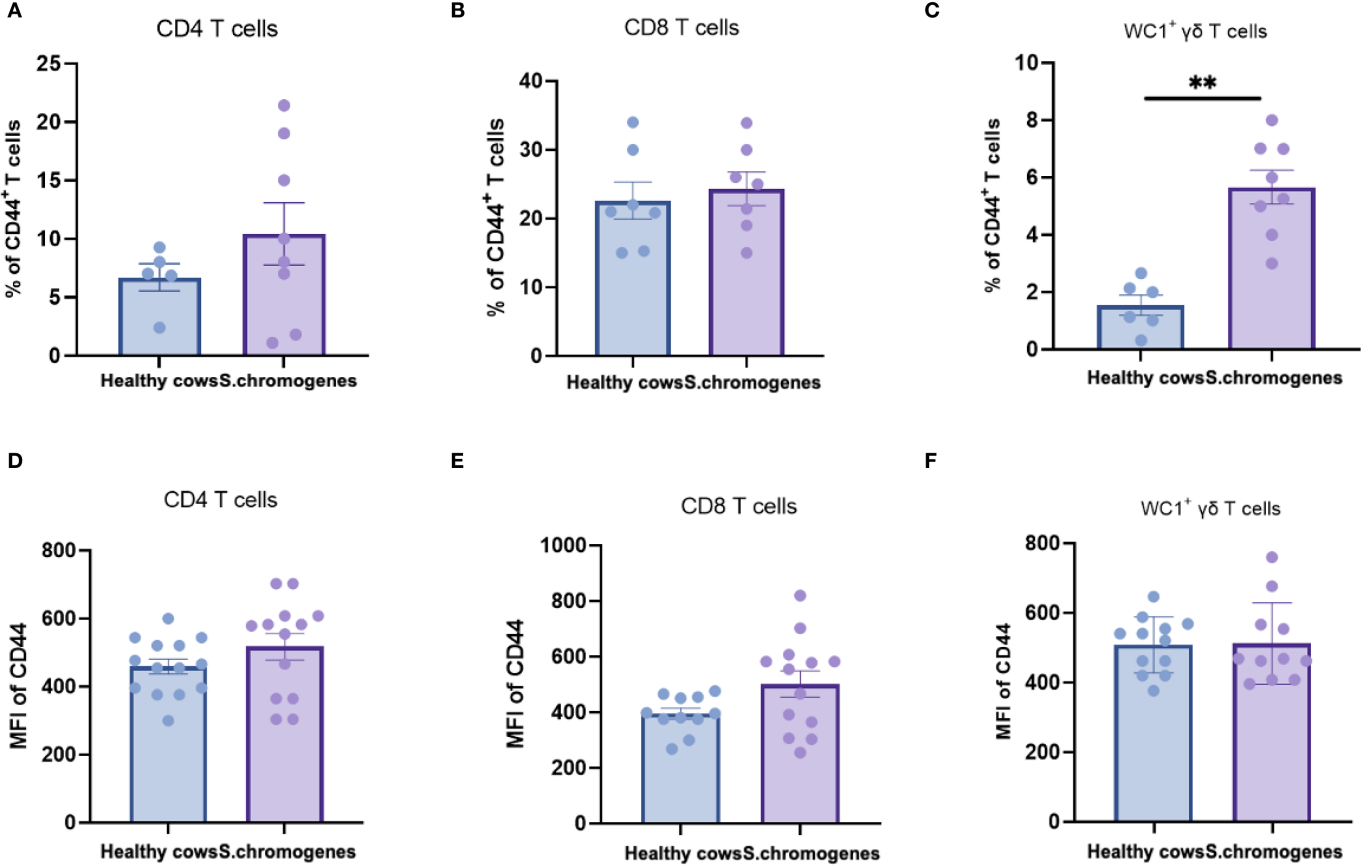
Difference of CD44 expression in different subsets of T cells (CD4^+^, CD8^+^, WC1^+^) in the milk of healthy and subclinical mastitis cows. Representative histograms show CD44 expression on CD4^+^CD8^-^ **(A)**, CD4^-^ CD8^+^ **(B)** T cells and WC1^+^ γδ T cells **(C)** from the milk. Bar graphs show MFI of CD44 on CD4^+^ **(D)**, CD8^+^ T cells **(E)** and WC1^+^ γδ T cells **(F)** from the two groups. Results were considered statistically significant when P < 0.01 (**).

As CD44 serves as a key activation marker for T-cell subsets, we analyzed its expression in peripheral blood from both groups. No significant differences were observed in CD44 expression frequencies or mean fluorescence intensity (MFI) among CD4^+^, CD8^+^, or WC1^+^ γδ T cells in *S. chromogenes* induced subclinical mastitis cows compared to healthy controls (**Figure 4**). However, In milk samples, WC1^+^ γδ T cells from affected cows exhibited significantly higher CD44 expression frequencies than healthy controls (**Figure 5**). This finding further substantiates WC1^+^ γδ T cells involvement in *S. chromogenes* triggered T-cell activation within the mammary microenvironment, indicating their crucial role in local antibacterial defense mechanisms during subclinical mastitis.

### 3.5 Transcriptional upregulation of multiple cytokines was observed in γδ T cells derived from *S. chromogenes*-induced subclinical mastitis in cows milk

**Figure 6:**
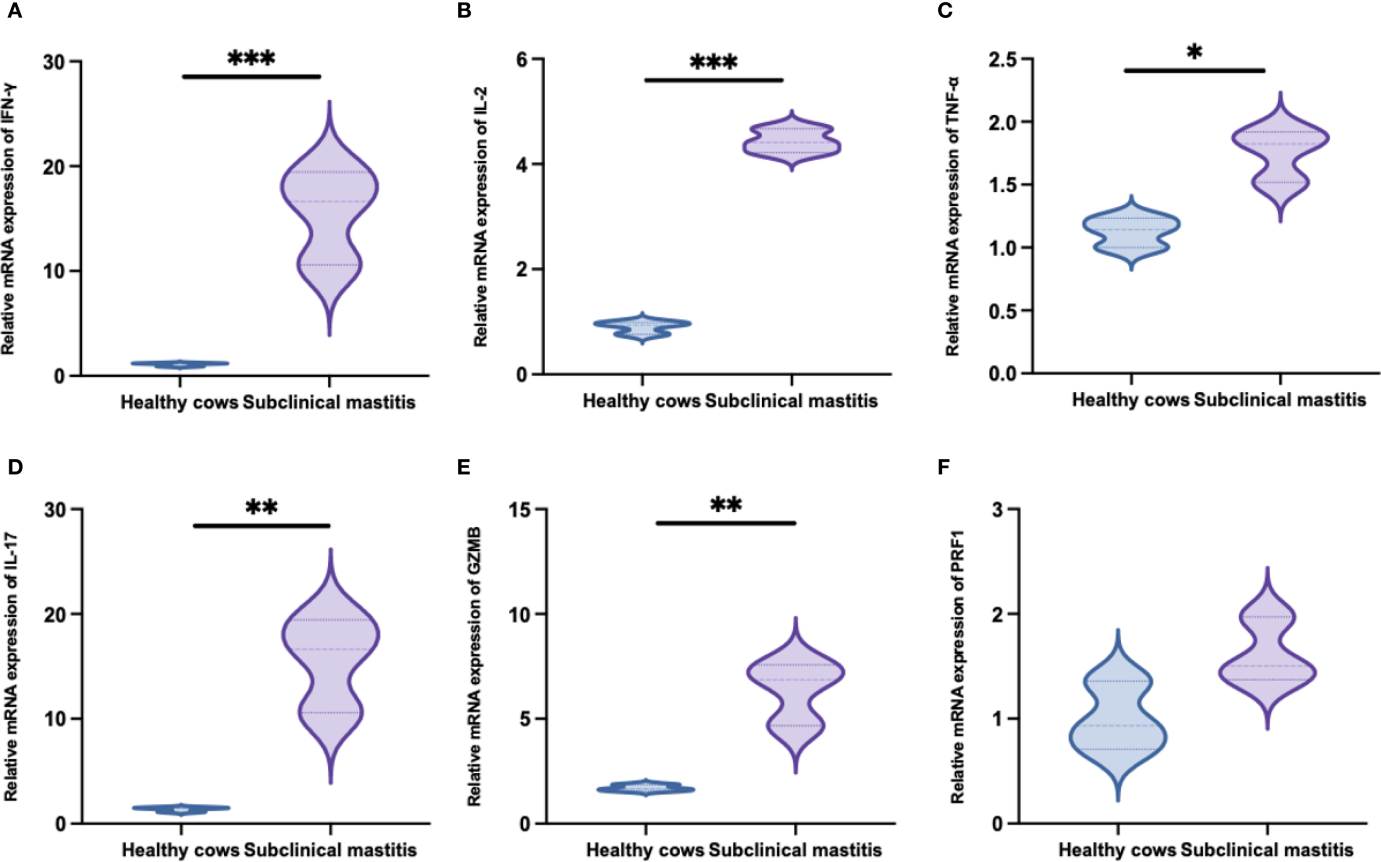
Transcription levels of immune-related genes following *Staphylococcus chromogenes* infection (mean fold difference compared with uninfected cows). The relative expression levels of IFN-γ **(A)**, IL-2 **(B)**, TNF-α **(C)**, IL-17 **(D)**, GZMB **(E)** and PRF1 **(F)** mRNA in WC1^+^ γδ T cells derived from milk. Results were considered statistically significant when P < 0.05 (*), P < 0.01 (**), or P < 0.001 (***).

To investigate the functional specificity of WC1^+^ γδ T-cells in *S. chromogenes* induced subclinical mastitis, transcriptional profiling was conducted. Results demonstrated infection-specific alterations in cytokine and effector molecule expression by milk WC1^+^ γδ T cells from affected cows (**Figure 6**). Notably, significant upregulation of IFN-γ, IL-2, TNF-α, and IL-17 expression was observed, suggesting WC1^+^ γδ T cells may recruit macrophages and neutrophils to mammary tissues through these cytokines while directly exerting cytotoxic effects on S. chromogenes-infected cells via granzyme B (GZMB) secretion.

## 4 Discussion

This study investigated 60 lactating dairy cows to identify subclinical mastitis pathogens through microbiological analysis. Mastitis significantly impacts dairy economics by reducing milk yield/quality and increasing veterinary antibiotic demand globally, with subclinical mastitis being the predominant form worldwide (18). This condition causing severe symptoms, economic losses, and premature culling, where *S. chromogenes* are the primary pathogens (19, 20). Using microbial culture and molecular techniques, we systematically characterized milk microbiome differences between healthy and mastitis-affected cows. *B. licheniformis* was more prevalent in healthy milk from farms using organic versus sand bedding, likely due to higher organic matter content (21). This highlights the necessity of rigorous bedding management—including prompt feces removal and regular microbial monitoring. *S. chromogenes* was particularly associated with subclinical mastitis cases (22). Phylogenetic analysis revealed that all seven YZU isolates shared >98% genetic identity with *S. chromogenes*, forming monophyletic clusters (YZU1-7) with distinctive positioning of YZU4. These seven strains were positioned between the subclinical strain MN535878.1 and the clinical mastitis strain MN314446.1. Based on the variability observed within our sample, the YZU4 strain demonstrates a modest degree of variability, indicating that the individual immune expression values for this strain do not markedly deviate from the norm within our population. There is no evidence of specific disparities in virulence or host adaptation for YZU4, nor does it appear to significantly influence the immune responses observed. Notably, this finding indicates that subclinical mastitis induced by *S. chromogenes* may arise from evolutionary changes within the bacterial strain itself, suggesting a potential association with subclinical disease progression and possible bacterial synergism (23). The immune response triggered by *S. chromogenes* induced subclinical mastitis in the host is highly noteworthy and warrants further investigation. Significant genetic differentiation from close relatives (e.g., *S. felis*) confirms its status as an independent evolutionary unit.

Next, we proceeded to explore the lymphocyte subsets found in PBMCs and milk from healthy cows and those with subclinical mastitis. It was discovered that the proportion of WC1^+^ γδ T cells in milk from cows with subclinical mastitis was significantly higher than in healthy cows, while this phenomenon was not observed in PBMCs, which aligns with previous reports (24). γδ T cells are considered innate-like T cells with a limited pre-activated phenotype and TCR repertoire. They can rapidly respond to infections or cytokine stimulation in a non-MHC-restricted manner, secrete a broad spectrum of cytokines, and exhibit direct cytotoxicity against infections and altered cells (25). γδ T cells constitute a small percentage of the peripheral lymphocyte repertoire in humans and rodents. In contrast, they represent a major lymphocyte subset in cattle, accounting for up to 60% of circulating T cells in calves (26). Therefore, γδ T cells may play a crucial role in bovine immune function. Up to 90% of these γδ T cells express a type I integral membrane protein known as WC1, which is one of the few differentiation antigens on γδ T cells (27). WC1^+^ γδ T cells constitute the predominant subset in circulation, whereas WC1^-^ γδ T cells are most abundant in the spleen, intestinal mucosa, and mesenteric lymph nodes. WC1^-^ γδ T cells are believed to play a role in immune surveillance and the maintenance of tissue quiescence, while WC1^+^ γδ T cells exhibit a more pronounced pro-inflammatory phenotype, capable of producing substantial amounts of IFN-γ in response to experimental challenges. Recent reports suggest that these populations may fulfill distinct functional roles in bovine immune responses, milk, as part of the systemic circulation of cows, is expected to exhibit proportions similar to those found in blood (28–31). Notably, the results indicate no significant differences in lymphocyte subsets between blood and milk, which can be attributed to the absence of overt inflammatory responses in cows with subclinical mastitis. Since γδ T cells complement αβ T cells in host defense by providing a rapid response before the full development of the αβ T cell response, acting as the first line of defense (32), does not trigger adaptive immunity in cows. The γδ T cells in cows with subclinical mastitis caused by *S. chromogenes* were found only in the mammary gland and did not enter the systemic circulation.

To better understand the host defense mechanisms of mastitis, we evaluated the changes in αβ T cells subsets observed in healthy dairy cows and those with subclinical mastitis induced by *S. chromogenes*. The findings indicate that the proportion of CD4^+^CD8^+^ double-positive (DP) T cells in milk is significantly reduced in cows with subclinical mastitis compared to healthy cows. CD8^+^ T cells, a subset of MHC class I-restricted T cells, serve as critical mediators of adaptive immunity (33). This population includes CD8^+^ cytotoxic T cells and CD8^+^ suppressor T cells. CD4^+^ T cells, also integral to adaptive immunity, help coordinate immune responses by stimulating other immune cells, such as macrophages, to combat infections (34). DP T cells are believed to represent a developmental stage in the thymus before maturing into CD8^+^ or CD4^+^ (single-positive) mature T cells (35). However, research in recent decades has successively confirmed the presence of functionally mature CD4^+^CD8^+^ T cells in the peripheral blood of humans (36), canines (37), and pigs (38). Early studies indicated that among various T cell subsets in pigs, CD4^+^CD8^+^ T cells are the primary population mediating interferon-gamma responses (39). Although the function of DP T cells has not been fully characterized, studies suggest they may play a role in adaptive immune responses to infections or tumors, as they tend to infiltrate sites of chronic infections or neoplastic lesions (40, 41). Surprisingly, our previous research found that although the proportion of CD4^+^CD8^+^ T cells in dairy cows is minimal, these cells exhibit diversity and pleiotropic activity, playing a crucial role in the immune response to bovine mastitis (42). Therefore, the observed decrease in the proportion of CD4^+^CD8^+^ T cells within mammary gland tissues affected by subclinical mastitis in this study may suggest an elevated risk of mastitis infection in dairy cows.

Activation of T cells is one of the initial stages of the host response to infection and migration of T cells from the circulation to areas of inflammation requires the progressive interaction of different adhesion molecules released by T cells (43). CD44 is a leukocyte adhesion molecule that interacts with components of the extracellular matrix and is thought to be required for extravasation of T cells from sites of inflammation (44). Our previous research findings indicate that the fluorescence intensity (MFI) of CD44 can be utilized to observe the immune response of T cells (45), and this adhesion molecule has been identified as a sensitive indicator of T cell activation (46). We determined the percentage of CD44^+^ T cells in the αβ or γδ T cells subsets of blood and milk from healthy and subclinical mastitis cows to assess activation of T cells. In addition, we determined the expression of CD44 molecules on T cells subsets from subclinical mastitis and healthy cows to study T cells subsets activation. We found that the percentage of activated WC1^+^ γδ T cells was significantly increased in the milk of cows with subclinical mastitis due to *S. chromogenes*. However, there was no difference in the percentage of CD4^+^ CD44^+^ and CD8^+^ CD44^+^ T cells in the milk of the two groups. While in blood there was no difference in both groups. Cow’s milk lymphocytes have been reported to express increased CD44 after receiving bacterial invasion within the milk, and it haLIns also been found that blood lymphocytes are recruited into the mammary gland (47). This suggests that the increased percentage of CD44 activation we detected in milk is a marker of bacterial invasion of the mammary gland of dairy cows, and that γδ T cells are selectively activated during host defense against *S. chromogenes* compared to αβ T cells. Our study further supports the notion that γδ T cells complement αβ T cell defense by giving a rapid response to infection caused by *S. chromogenes* before the αβ T cell response is fully formed, the first line of line of defense.

Subsequently, we measured the mRNA expression levels of IFN-γ, IL-2, TNF-α, IL-17, GZMB, and PRF1 in WC1^+^ γδ T cells from milk. The results demonstrated that, with the exception of PRF1, cows with subclinical mastitis induced by *S. chromogenes* exhibited significantly higher expression levels of these five cytokines compared to healthy cows. Notably, as the prototypical non-conventional lymphocytes, γδ T cells exhibit diverse and indispensable functions in tissue homeostasis and immune responses against pathogens or tumors (48). The reported roles of γδ T cells during infection can be primarily categorized into the following aspects: γδ T cells serve as a crucial early source of cytokines during infection. On one hand, they secrete cytokines to modulate the inflammatory microenvironment, particularly IFN-γ, IL-2, and IL-17, which play pivotal roles in preventing mastitis and exert direct effects on tissue and downstream adaptive immune responses (49, 50). Studies have indicated that γδ T cells exhibit Th1-like helper functions, enhancing cell-mediated anti-infective immunity. They play a significant role in initiating inflammatory responses and regulating the expansion and recruitment of neutrophils and monocytes (51). Additionally, γδ T cells possess unique advantages, such as the ability to induce immature dendritic cells to express CD86 and MHC class I molecules via TNF-α secretion, MHC-independent antigen recognition, and direct tumor cell lysis (52). On the other hand, γδ T cells exhibit cytotoxic effects by releasing cytotoxic molecules such as perforin (PRF1) and granzyme B (GZMB), enabling direct killing of target cells infected with intracellular bacteria or viruses (53). In the milk of subclinical mastitis in this study, WC1^+^ γδ T cells exhibited significant upregulation of IFN-γ, IL-2, TNF-α, IL-17, and GZMB. Based on published literature, γδ T cells play a crucial role in defending against *Staphylococcus aureus* infections. *S. chromogenes* is an opportunistic pathogen frequently found in the skin and mammary glands of cattle, capable of inducing subclinical mastitis in dairy cows only under specific conditions. Therefore, in this experiment, the inflammatory response triggered by *S. chromogenes* was mediated by γδ T cells, which constitute the host’s first line of defense. Presently, our study has limited its qPCR analysis to cytokines and cytotoxic molecules within γδ T cells, while functional assessments such as intracellular cytokine secretion assays and cytotoxicity measurements remain to be further substantiated. In future research endeavors, we intend to validate these factors at the protein level. Based on this experimental study, it is feasible to propose that γδ T cells within the mammary gland exhibit a rapid response, functioning as the first line of defense, effectively mediating immune responses in cows suffering from subclinical mastitis caused by *S.chromogenes*. This suggests that γδ T cells could potentially serve as a biomarker for immunoregulation in subclinical bovine mastitis, enabling rapid and cost-effective diagnostic assays for the condition.

In summary, due to the limitations of our research subject—cattle—and the insufficient availability of flow cytometry antibodies, we were unable to conduct a comprehensive analysis of bovine lymphocyte subsets. This study investigated the distribution characteristics of lymphocyte subsets and the expression of cytokines secreted by γδ T cells in blood and milk samples from dairy cows with subclinical mastitis induced by *S. chromogenes* and their healthy counterparts. The results demonstrated that γδ T cells in the mammary glands of infected cows were significantly elevated, with a marked increase in the relative expression levels of secreted cytokines, while the number of CD4^+^ CD8^+^ T cells significantly decreased, exhibiting a unique activation pattern. Due to potential limitations in statistical power resulting from the final sample size (n=20 per group), our primary objective was to exclusively investigate the effects of *S.chromogenes*, necessitating the exclusion of cattle with mixed infections as co-infections could confound the observed outcomes. Although the sample size may constrain the ability to detect subtle effects, this study prioritized ensuring homogeneity in infection status. Nevertheless, subgroup analyses of excluded samples and their potential confounding effects, given the requirements of the study, will be addressed in future research by expanding the cohort to accommodate these considerations. Based on this experiment, although *S. chromogenes* itself exhibits low pathogenicity, γδ T cells serve as the first line of defense, effectively mediating the immune response to subclinical mastitis caused by this bacterium. However, the continuous secretion of cytokines by γδ T cells leads to T cell exhaustion within the mammary gland. When bacterial synergism occurs, it breaches the primary barrier of the mammary gland, thereby increasing the risk of clinical mastitis in dairy cows. The decline in the proportion of CD4^+^ CD8^+^ T cells also elevates the risk of infection, as T cell exhaustion may impair their normal function. In the future, we will further investigate the lymphocyte subsets in cattle to obtain more definitive research findings on the immune response associated with subclinical mastitis in cows.

## Data Availability

The datasets presented in this study can be found in online repositories. The names of the repository/repositories and accession number(s) can be found in the article/**Supplementary Material**.
