# Supplementary material for: Characterization of T-cell immune responses against Staphylococcus chromogenes naturally-induced subclinical mastitis in dairy cows
Source: Front Immunol. 2025 Oct 10;16:1679963. doi: 10.3389/fimmu.2025.1679963 (PMC12549283; doi:10.3389/fimmu.2025.1679963)
Supplement: Supplementary file 1 [file DataSheet1.docx]

Supplementary Table 1. The Specific primer sequences of IFN-γ, IL-2, TNF-α, IL-17, GZMB and PRF1.

| **Primer name** | **Primer sequence** |
| --- | --- |
| IFN-γ-F | CACCACTTCGAAACCTGGGA |
| IFN-γ- R | TGTAGGCCCCAGTGAGTTCT |
| IL-2-F | GCAGCAACAGTCTTACCT |
| IL-2-R | ACAGGACTTGGGAGGTAT |
| TNF-α-F | ATGTCTCAGCCTCTTCTCATTC |
| TNF-α-R | GCTTGTCACTCGAATTTTGAGA |
| IL-17-F | AGGGACAGAGTCAAGTGCAG |
| IL-17-R | TGGCTCTGGTCCCCAAAT |
| GZMB-F | ACAGTTTGTTCCTCGTGGC |
| GZMB-R | CCATTCACGTCGTCCTTAT |
| PRF1-F | CAAGTTTCCATTCCGCTTC |
| PRF1-R | CTGGTGCAGCTCTCATATT |

Supplementary Table 2. Antibodies used for flow cytometry in this study.

| **Marker** | **Clone** | **Isotype** | **Conjugate** | **Conjugate type** | **Labeling strategy** |
| --- | --- | --- | --- | --- | --- |
| CD3 | MM1A | IgG_1_ | PerCP-Cy5.5 | Secondary antibody | Kingfisher |
| WC1 | CC15 | IgG2a | FITC | Direct conjugate | Bio-Rad |
| CD4 | CC8 | IgG2a | Alexa Fluor 647 | Direct conjugate | Bio-Rad |
| CD8 | CC63 | IgG2a | PE | Direct conjugate | Bio-Rad |
| CD44 | IMC | IgG2b, κ | PrestoBlue | Direct conjugate | BioLegend |

Supplementary Figure 1. Gating strategy for defining bovine T cell subsets.


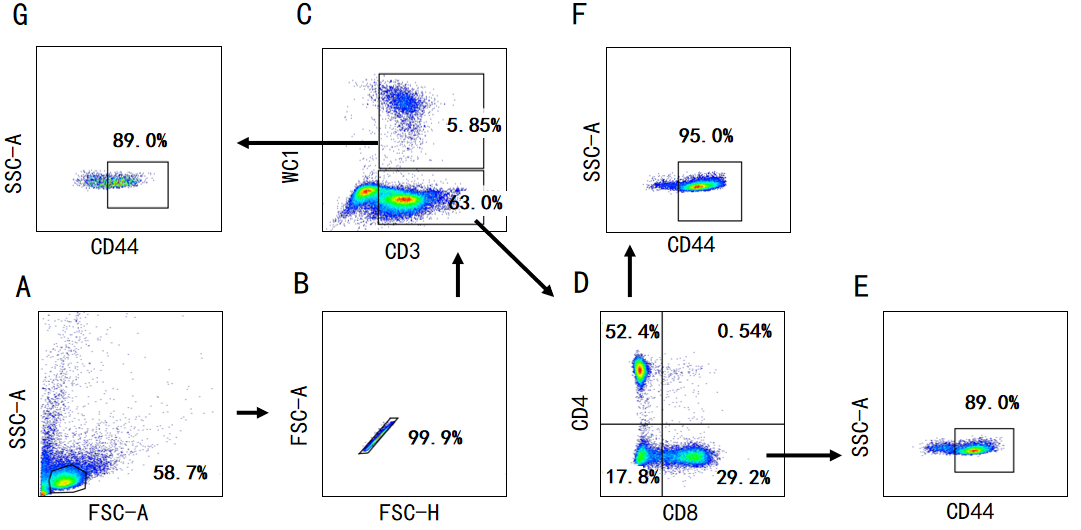


Monocytes were isolated from cow blood and milk and stained with antibody mixtures (CD3, CD4, CD8, WC1, CD44). Lymphocytes were selected with FSC and SSC (A), and then single cells were gated with FSC-A and FSC-H (B). CD3 and WC1, WC1^−^ CD3^+^ T cells and WC1^+^ γδ T cells were identified by exporting them (C). CD4^+^ CD8^−^, CD8^+^ CD4^−^, and CD8^+^ CD4^+^ were identified by exporting CD4 and CD8 in WC1^−^CD3^+^ T cells, respectively (D). CD4^-^CD8^+^ T cells contain CD44^+^ (E), CD4^+^ CD8^−^ T cells contain CD44^+^ (F), WC1^+^ γδ T cells contain CD44^+^ (G).

Supplementary Figure 2. Gating strategy for WC1^+^ γδ T Cell Sorting


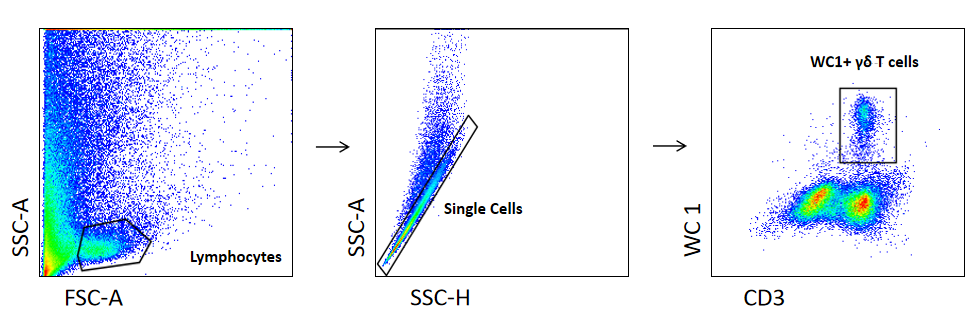


Lymphocytes were identified via their size and granularity using FSC-A in conjunction with SSC-A. Individual lymphocytes were further delineated and gated through the use of SSC-A in combination with SSC-H. WC1+ cells were subsequently identified, gated, and subjected to gating procedures.
